# Dose‐escalated radiation therapy is associated with better overall survival in patients with bone metastases from solid tumors: a propensity score‐matched study

**DOI:** 10.1002/cam4.1150

**Published:** 2017-08-15

**Authors:** Yung‐Chih Chou, Chien‐Yu Lin, Ping‐Ching Pai, Chen‐Kan Tseng, Cheng‐En Hsieh, Kai‐Ping Chang, Cheng‐Lung Hsu, Chun‐Ta Liao, Chun‐Chieh Wang, Shy‐Chyi Chin, Tzu‐Chen Yen, Tsung‐Ying Ho, Ji‐Hong Hong, Kin‐Fong Lei, Joseph Tung‐Chieh Chang, Ngan‐Ming Tsang

**Affiliations:** ^1^ Department of Radiation Oncology Chang Gung Memorial Hospital and University at Lin‐Kou Taoyuan Taiwan; ^2^ Department of Otolaryngology, Head and Neck Surgery Chang Gung Memorial Hospital and Chang Gung University at Lin‐Kou Taoyuan Taiwan; ^3^ Division of Hematology‐Oncology Department of Internal Medicine Chang Gung Memorial Hospital Chang Gung University Taoyuan Taiwan; ^4^ Department of Otorhinolaryngology, Head and Neck Surgery Chang Gung Memorial Hospital and Chang Gung University Taoyuan Taiwan; ^5^ Department of Medical Imaging and Intervention Linkou Medical Center Chang Gung Memorial Hospital Taoyuan Taiwan; ^6^ Department of Nuclear Medicine and Molecular Imaging Center Chang Gung Memorial Hospital and Chang Gung University Taoyuan Taiwan; ^7^ Graduate Institute of Medical Mechatronics Chang Gung University Taoyuan Taiwan; ^8^ School of Traditional Chinese Medicine Chang Gung University Taoyuan Taiwan

**Keywords:** Bone metastasis, dose escalation, overall survival, solid tumors

## Abstract

We aimed to compare the overall survival (OS) of patients with bone metastases (BM) from solid tumors after standard‐dose radiotherapy ([RT]; 30 Gy administered in 10 fractions; EQD_2Gy_ = 32.5 Gy) and dose‐escalated RT (EQD_2Gy_ > 32.5 Gy). We retrospectively reviewed the clinical charts of 1795 patients (median age, 62.3 years; age range, 18–96 years) with BM from solid tumors who were referred for RT to our institute between 2000 and 2013. These patients were assigned to the standard‐dose (*n* = 1125; 63%) and dose‐escalated (*n* = 670; 37%) RT groups. OS, estimated as the duration between the first RT session and death, served as the main outcome measure. The dose‐escalated RT group had a significantly better OS than the standard‐dose RT group (*P* = 0.000). After allowing potential confounders in multivariate analysis, the RT dose retained its independent association with OS (hazard ratio [HR], 0.837; 95% confidence interval [CI], 0.753–0.929, *P* = 0.001). After propensity score matching of the baseline characteristics of both groups, RT dose retained its independent association with OS (HR, 0.887; 95% CI, 0.737–0.951; *P* = 0.011) on multivariate analysis. Dose‐escalated RT exerted more favorable effects on OS in patients with non‐lung cancer, those without multiple metastases, those without symptoms, and those with favorable prognosis. Dose‐escalated RT was significantly associated with better OS in patients with BM from solid malignancies, particularly among those with non‐lung cancer, those without multiple metastases, those without symptoms, and those with favorable prognosis.

## Introduction

The bone is the one of the most frequently involved site of metastasis from solid tumors 1. Bone metastasis (BM) is indicative of a short‐term prognosis, and the median survival from the time of diagnosis is several months 2. The development of skeletal‐related events (such as spinal cord compression and pathologic fractures) from BM leads to considerable morbidity and mortality 3. In such cases, the use of multidisciplinary interventions aimed at reducing pain, increasing bone stability, preserving function, and improving local tumor control has become increasingly common.

In theory, improved disease control can prolong survival in patients with cancer. Systemic therapies (e.g., chemotherapy, hormonal therapy, or targeted therapy) are known to improve overall survival (OS) in certain patients with distant metastases 4, 5. The surgical management of isolated BM is also feasible and improves survival outcomes 6, 7. Reports indicate that radiotherapy (RT) dose escalation improves local disease control in patients with BM 8, 9. However, it is unclear whether RT dose escalation is still associated with better OS in patients with BM.

The linear‐quadratic radiobiological model enables the comparison of different fractionation schedules by calculating the equivalent dose in 2 Gy fractions (EQD_2Gy_). Using this linear‐quadratic model, the shape of the survival curve can be determined via the *α*/*β* ratio, which has units of radiation dose. In the present study, assuming an *α*/*β* ratio of 10 Gy for clearing tumor cells, we aimed to compare the OS after standard‐dose RT (30 Gy administered over 10 fractions; EQD_2Gy_ = 32.5 Gy) and dose‐escalated RT (EQD_2Gy_ > 32.5 Gy) for patients with BM from solid tumors.

## Materials and Methods

### Study patients

Between January 2000 and December 2013, we retrospectively reviewed the clinical charts of patients with solid malignancies who were referred for RT to the Chang Gung Memorial Hospital. All participants had a histology‐proven diagnosis of cancer, except for some patients with hepatocellular carcinoma, for whom imaging evidence alone was considered to be sufficient 10. The exclusion criteria were as follows: history of palliative RT for metastases located in anatomical sites other than the bone; EQD_2Gy_ < 32.5 Gy; unavailability of official pathological reports; unavailability of baseline characteristics (e.g., education level or employment status); patient age <18 years; and loss to follow‐up within 6 months after RT completion. All data were collected by a radiation oncologist and an experienced nurse. The study protocol was approved by the Institutional Review Board of the Chang Gung Memorial Hospital (IRB 201601358B0). Due to the retrospective nature of the study, the need for informed consent was waived.

### Definitions of the study variables

The duration of RT was defined as the time interval between the first and the final RT session. The field area was calculated by multiplying the maximum field length per field width. Patient age was recorded during the first visit to the radiation oncologist. The patients with symptoms of BM before RT were categorized into three groups: those without symptoms, those with pain, and those with neurological deficits. The time for metastasis was defined as the time interval between the initial primary cancer diagnosis and the identification of distant metastases from the same tumor. For the purpose of analysis, this value was categorized as >1 year or ≤1 year. Metastases to the bone were dichotomized as spinal or nonspinal metastases. Multiple metastases were considered when ≥2 different organs were involved, or if different parts of the skeleton were affected (e.g., sternum and sacrum), or multiple spinal metastases were present that required a treatment field >12.5 cm in size. The location of the primary tumor was categorized as lung cancer and non‐lung cancer (including urinary tract cancer, breast cancer, gastrointestinal cancer, and other cancers). Systemic treatments were analyzed, starting from 1 month before RT to the date of the last follow‐up. The detailed definitions of the study variables have been previously reported 11. In brief, the performance status was determined using the Eastern Cooperative Oncologic Group (ECOG) scale. The presence of comorbidities (dichotomized as yes/no) was assessed, using the Charlson Comorbidity Index 12. Employment status was categorized as “unemployed,” “low‐wage employed,” or “high‐wage employed,” according to the Registrar General's Social Class (RGSC) scheme, with slight modifications. The education level was classified as low (elementary school and below) or high (junior high school and above). The patients’ residence was coded as rural or urban (population density >800 persons per square kilometer). As per the US Centers for Disease Control and Prevention (CDC) classification system 13, cigarette smoking was dichotomized as yes (subjects who smoked ≥100 cigarettes in their lifetime) or no (subjects who smoked <100 cigarettes in their lifetime and no current smoking status). Similarly, alcohol consumption (current and former drinkers vs. never drinkers) and betel quid chewing (current and former chewers vs. never chewers) were treated as dichotomized variables.

### Statistical analysis

OS served as the main outcome measure and was calculated as the time interval (in months) from the date of the first RT session to the date of death. Local control was defined as “no new tumors” in the previous radiation field. The diagnosis of local failure of BM was confirmed, using computed tomography or magnetic resonance imaging. The differences between the standard‐dose RT and dose‐escalated RT groups were assessed using Student's *t*‐tests (continuous variables) or *χ* ^2^ tests (categorical data). Survival curves were plotted with the Kaplan–Meier method, and compared with the log‐rank test. Multivariate Cox proportional hazards regression analyses were used to identify the independent predictors of OS. Results were expressed as hazard ratios (HRs) with 95% confidence intervals (CIs). The heterogeneity of the treatment effects in subgroup analyses was assessed by comparing the baseline characteristics identified as significant prognostic factors in the entire study cohort (i.e., sex, performance status, primary cancer, time to metastases, presence of multiple metastases, systemic therapy, education level, and betel quid chewing). In all analyses, two‐tailed *P* < 0.05 was considered statistically significant. The propensity score was estimated via logistic regression, using the dependent variable in the two groups. All data were analyzed using the SPSS 24.0 software package (IBM Corporation, Armonk, NY). Propensity score matching was performed with the R program, version 3.03, in the MatchIt package (R Foundation for Statistical Computing, Vienna, Austria).

## Results

Between 2000 and 2013, we identified 7557 patients with distant metastases from solid tumors who were treated with RT. Patients with a history of palliative RT to metastases located in anatomical sites other than the bone (*n* = 3650), EQD_2Gy_ < 32.5 Gy (*n* = 2015), unavailable official pathological reports (*n* = 12), unavailable baseline characteristics (*n* = 39), and age <18 years (*n* = 33), as well as those lost to follow‐up within 6 months after RT completion (*n* = 13) were excluded. Consequently, 1795 patients (median age, 62.3 years; age range, 18–96 years) were included in the present analysis. With regard to the primary cancer location, 607 patients (33.8%) had lung cancer, 296 (16.5%) had urinary tract cancer, 242 (13.5%) had breast cancer, and 650 (36.2%) had other types of solid tumors. Table S1 illustrates the relationship between RT doses and the primary tumor location. The median time between primary tumor diagnosis and the identification of distant metastases was 5.28 months (range, 0–17.56 years). A total of 1125 (63%) and 670 (37%) patients were assigned to the standard‐dose and dose‐escalated RT groups, respectively. Table 1 summarizes the general baseline characteristics of the study participants according to the RT group. The mean EQD_2Gy_ values for the standard‐dose and dose‐escalated RT groups were 32.5 Gy and 40.4 Gy, respectively. The dose‐escalated schemes varied widely, although the most common dose‐escalated RT schemes were 2.5 Gy × 14 fractions (13.4%), 3 Gy × 11 fractions (12.8%), 3 Gy × 12 fractions (9.3%), 4 Gy × 7 fractions (8.2%), 5 Gy × 6 fractions (6%), and 2 Gy × 20 fractions (4.9%). The mean RT duration in the standard‐dose and dose‐escalated RT groups was 14.3 days (standard deviation, 2.9 days) and 19.6 days (standard deviation, 9.3 days), respectively.

**Table 1:** Baseline characteristics according to the radiotherapy dose of patients with bone metastases from solid tumors in the cohort study and after propensity score matching

	Entire cohort (*n* = 1795)			Propensity score matching (*n* = 1340)		
	Standard‐dose RT *n* = 1125 (63%)	Dose‐escalated RT *n* = 670 (50%)	*P*	Standard‐dose RT *n* = 670 (50%)	Dose‐escalated RT *n* = 670 (50%)	*P*
EQD_2Gy_						
Mean±SD	32.5 ± 0	40.4 ± 7.5	<0.001	32.5 ± 0	40.4 ± 7.5	<0.001
Median (range)	32.5 (0)	38.1 (32.6–73.2)		32.5 (0)	38.1 (32.6–73.2)	
Dose per fraction (cGy)						
Mean±SD	300 ± 0	317.13 ± 104.9	<0.001	300 ± 0	317.13 ± 104.9	<0.001
Median (range)	300	300 (167–800)		300	300 (167–800)	
RT duration (days)						
Mean±SD	14.3 ± 2.9	19.6 ± 9.3	<0.001	14.4 ± 3.3	19.6 ± 9.3	<0.001
Age (years)						
Mean±SD	62.4 ± 12.6	59.6 ± 12.9	<0.001	59.7 ± 12.0	59.6 ± 12.9	0.771
<60, *n* (%)	454 (40.4)	340 (50.8)	<0.001	332 (49.5)	340 (50.8)	0.672
≥60, *n* (%)	671 (59.6)	330 (49.2)		338 (50.4)	330 (49.2)	
Symptoms						
Absence of symptoms, *n* (%)	72 (6.4)	61 (9.0)	0.013	72 (10.7)	61 (9.0)	0.776
Pain, *n* (%)	735 (65.3)	469 (70)		467 (69.8)	469 (70)	
Neurological deficits, *n* (%)	318 (28.3)	141 (21.0)		131 (19.5)	141 (21.0)	
Time to metastases (years)						
Mean±SD	1.06 ± 2.2	1.58 ± 2.7	<0.001	1.62 ± 2.7	1.58 ± 2.7	0.388
<1 year, *n* (%)	842 (74.8)	418 (62.4)	<0.001	413 (61.8)	418 (62.4)	0.803
≥1 year, *n* (%)	283 (25.2)	252 (37.6)		257 (38.2)	252 (37.6)	
Sex						
Male, *n* (%)	475 (42.2)	300 (44.8)	0.343	289 (43.1)	300 (44.8)	0.337
Female, *n* (%)	650 (57.8)	370 (55.2)		381 (56.9)	370 (55.2)	
Performance status						
ECOG 0–1, *n* (%)	515 (45.8)	354 (52.8)	0.002	352 (52.4)	354 (52.8)	0.878
ECOG 2–4, *n* (%)	610 (54.2)	316 (47.2)		318 (47.6)	316 (47.2)	
Site of bone metastases						
Spinal lesions, *n* (%)	863 (76.7)	368 (54.9)	<0.001	408 (60.8)	368 (54.9)	0.031
Nonspinal lesions, *n* (%)	262 (23.3)	302 (45.1)		262 (39.2)	302 (45.1)	
Metastases to >1 site						
No, *n* (%)	270 (24)	168 (25.2)	0.518	169 (25.2)	168 (25.2)	0.903
Yes, *n* (%)	855 (76)	502 (74.8)		501 (74.8)	502 (74.8)	
Location of primary cancer						
Lung, *n* (%)	443 (39.5)	164 (24.5)	<0.001	156 (23.3)	164 (24.5)	0.372
Urinary tract, *n* (%)	193 (17.2)	103 (15.4)		100 (14.9)	103 (15.4)	
Breast, *n* (%)	122 (10.8)	120 (17.9)		115 (17.1)	120 (17.9)	
Other primary cancer location, *n* (%)	367 (32.6)	283 (42.2)		309 (44.7)	283 (42.2)	
Systemic therapy						
No, *n* (%)	642 (57.1)	355 (52.9)		349 (52.1)	355 (52.9)	0.836
Yes, *n* (%)	483 (42.9)	315 (47.1)	0.132	321 (47.9)	315 (47.1)	
Comorbidities						
No, *n* (%)	699 (62.1)	396 (59)		403 (60.1)	396 (59)	0.798
Yes, *n* (%)	426 (37.9)	274 (41)	0.284	267 (39.9)	274 (41)	
Employment status						
High‐wage employed, *n* (%)	233 (20.7)	154 (23.0)		150 (22.4)	154 (23.0)	0.821
Low‐wage employed, *n* (%)	360 (32)	193 (28.8)	0.299	205 (30.6)	193 (28.8)	
Unemployed, *n* (%)	532 (47.3)	323 (48.2)		315 (47.0)	323 (48.2)	
Education level						
None/primary	674 (59.9%)	347 (51.7%)		334 (49.9%)	347 (51.7%)	0.531
Higher	451 (40.1%)	323 (48.3%)	0.002	336 (50.1%)	323 (48.3%)	
Place of residence						
Urban, *n* (%)	651 (57.8)	382 (57.1)		391 (58.2)	382 (57.1)	0.648
Rural, *n* (%)	474 (42.2)	288 (42.9)	0.912	279 (41.8)	288 (42.9)	
Cigarette smoking						
No, *n* (%)	675 (60)	401 (59.9)		385 (57.4)	401 (59.9)	0.442
Yes, *n* (%)	450 (40)	269 (40.1)	0.849	285 (42.6)	269 (40.1)	
Betel quid chewing						
No, *n* (%)	1008 (89.6)	584 (87.2)		569 (84.9)	584 (87.2)	0.401
Yes, *n* (%)	117 (10.4)	86 (12.8)	0.322	101 (15.1)	86 (12.8)	
Alcohol drinking						
No, *n* (%)	864 (76.8)	494 (73.8)		477 (71.2)	494 (73.8)	0.291
Yes, *n* (%)	261 (23.2)	176 (26.2)	0.127	193 (28.8)	176 (26.2)	
Propensity score						
Mean±SD	0.672 ± 0.146	0.586 ± 0.157	<0.00	0.599 ± 0.115	0.586 ± 0.157	0.264
OS, months						
Median (range)	5.75 (5.17–6.34)	8.48 (7.16–9.80)	<0.0010	6.27 (5.39–7.36)	8.48 (7.16–9.80)	<0.0010
1‐year OS rate, %	30.4	40.7	<0.00	34.2	40.7	<0.00
2‐year OS rate, %	16.0	23.7		19.1	23.7	
Local control, months						
1‐year LC rate, %	96.7	98.3	0.007			
2‐year LC rate, %	90.3	95.2	0.014			
4‐year LC rate, %	81.5	86.4	0.031			

LC, Local control; OS, overall survival; RT, radiotherapy.

### OS and local control

**Figure 1:**
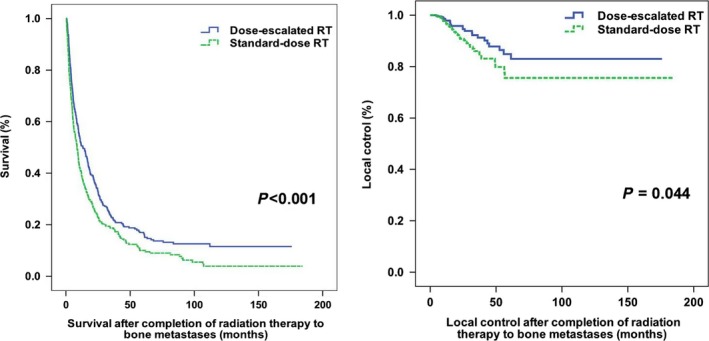
Kaplan–Meier plots of overall survival and local control according to the radiotherapy (RT) dose in patients with bone metastases from solid tumors.

The median follow‐up duration for surviving patients was 46.7 months (range, 7.5–184.4 months). A total of 1668 patients died at follow‐up. The 1‐ and 2‐year OS rates in the entire study cohort were 34.2% and 18.9%, respectively (median OS, 6.54 months; range, 6 days to 184.4 months). The dose‐escalated RT group had a significantly better OS and local control than the standard‐dose RT group (*P* = 0.001, *P* = 0.044; Figure 1). Several variables were found to be significantly associated with OS on univariate analysis (Table 2). After allowing potential confounders on multivariate analysis, the RT dose retained its independent association with OS (HR, 0.837, 95% CI = 0.753–0.929, *P* = 0.001; Table 2).

**Table 2:** Univariate and multivariate Cox regression analyses of factors associated with overall survival in patients with bone metastases from solid tumors in the cohort study and after propensity score matching

	Entire cohort (*n* = 1795)				Propensity score matching (*n* = 1340)			
	Univariate analysis		Multivariate analysis		Univariate analysis		Multivariate analysis	
	Overall survival HR (95% CI)	*P*	Overall survival HR (95% CI)	*P*	Overall survival HR (95% CI)	*P*	Overall survival HR (95% CI)	*P*
EQD_2Gy_ (>32.5 Gy vs. = 32.5 Gy)	0.758 (0.686–0.837)	0.000	0.837 (0.753–0.929)	0.001	0.813 (0.758–0.914)	0.000	0.887 (0.737–0.951)	0.011
Age (≥60 years vs. <60 years)	1.297 (1.176–1.429)	0.000	0.995 (0.871–1.137)	0.942	1.186 (1.052–1.312)	0.003	0.972 (0.846–1.145)	0.837
Sex (female vs. male)	0.721 (0.654–0.795)	0.000	1.205 (1.103–1.411)	0.003	1.326 (1.118–1.497)	0.000	1.185 (1.088–1.375)	0.013
Symptoms								
Absence of symptomsversus pain	0.758 (0.620–0.928)	0.007	0.790 (0.644–0.968)	0.023	0.864 (0.694–1.038)	0.061	0.910 (0.767–1.186)	0.117
Absence of symptomsversus Neurologicaldeficits	0.809 (0.714–0.916)	0.001	0.833 (0.735–0.944)	0.004	0.805 (0.702–0.911)	0.000	0.813 (0.714–0.929)	0.002
Site of bonemetastases (spinalvs. nonspinal)	1.152 (1.038–1.278)	0.008	1.013 (0.900–1.141)	0.803	1.026 (0.916–1.150)	0.652	1.004 (0.892–1.132)	0.943
Performance status(ECOG 0/1 vs. ≥2)	0.795 (0.722–0.876)	0.000	0.738 (0.663–0.821)	0.000	0.822 (0.731–0.924)	0.001	0.777 (0.692–0.872)	0.000
Location of primary cancer								
Urinary tract versuslung	1.156 (1.031–1.296)	0.013	0.932 (0.886–0.973)	0.023	1.117 (0.971–1.271)	0.192	1.133 (0.977–1.316)	0.201
Breast versus lung	0.695 (0.602–0.802)	0.000	0.601 (0.526–0.707)	0.000	0.675 (0.510–0.790)	0.000	0.611 (0.502–0.745)	0.000
Others versus lung	0.456 (0.388–0.536)	0.000	0.591 (0.497–0.716)	0.000	0.466 (0.394–0.550)	0.000	0.589 (0.486–0.712)	0.000
Time to metastases(>1 year vs. ≤1 year)	0.735 (0.661–0.818)	0.000	1.112 (0.981–1.260)	0.098	0.712 (0.641–0.812)	0.000	0.882 (0.722–0.974)	0.039
Metastases to >1 site(no vs. yes)	0.572 (0.507–0.645)	0.000	0.568 (0.501–0.643)	0.000	0.509 (0.436–0.671)	0.000	0.489 (0.412–0.644)	0.000
Comorbidities (yes vs.no)	1.029 (0.932–1.135)	0.571	1.045 (0.933–1.171)	0.446	0.956 (0.814–1.107)	0.376	1.009 (0.952–1.137)	0.783
Systemic therapy (yesvs. no)	0.695 (0.631–0.766)	0.000	0.676 (0.604–0.756)	0.000	0.688 (0.617–0.751)	0.000	0.653 (0.601–0.718)	0.000
Employment status								
Low‐wage employedversus high‐wageemployed	1.280 (1.117–1.467)	0.000	0.910 (0.781–1.061)	0.229	0.937 (0.887–1.135)	0.246	0.978 (0.899–1.103)	0.456
Unemployed versushigh‐wageemployed	1.142 (1.007–1.295)	0.039	1.019 (0.899–1.168)	0.788	1.158 (0.987–1.293)	0.071	1.012 (0.872–1.203)	0.569
Education level (highvs. low)	0.768 (0.696–0.846)	0.000	1.101 (0.983–1.134)	0.131	0.811 (0.727–0.909)	0.000	1.086 (0.954–1.235)	0.213
Place of residence(rural vs. urban)	1.040 (0.944–1.146)	0.423	1.021 (0.915–1.138)	0.714	1.061 (0.949–1.187)	0.372	0.971 (0.864–1.091)	0.639
Cigarette smoking(yes vs. no)	1.320 (1.197–1.456)	0.000	0.946 (0.819–1.093)	0.450	1.345 (1.202–1.506)	0.000	0.968 (0.823–1.137)	0.732
Betel quid chewing(yes vs. no)	1.407 (1.215–1.630)	0.000	0.819 (0.679–0.988)	0.037	1.387 (1.165–1.647)	0.000	1.241 (1.016–1.515)	0.019
Alcohol drinking(yes vs. no)	1.263 (1.131–1.411)	0.000	1.010 (0.871–1.171)	0.0894	1.326 (1.170–1.503)	0.000	1.022 (0.870–1.135)	0.697

RT, radiotherapy; HR, hazard ratio; CI, confidence interval; EQD_2Gy_, equivalent dose in 2 Gy fractions; ECOG, Eastern Cooperative Oncology Group.

### Propensity score matching

To minimize the selection biases in our retrospective cohort, we used propensity score matching to balance the standard‐dose and dose‐escalated RT groups. We focused on the baseline significant differences at the *P* ≤ 0.1 level (Table 1). We found that the dose‐escalated RT patients were significantly more likely to be younger (*P* < 0.001); not have symptoms; have less neurological deficits (*P* = 0.013), longer time to metastases (*P* < 0.001), better performance status (*P* = 0.002), less spinal lesions (*P* < 0.001), and breast cancer as the primary tumor (*P* < 0.001); and were significantly less likely to have lung cancer as the primary tumor (*P* < 0.001). Therefore, we selected age, time to metastases, performance status, initial symptoms, and primary cancer type as the independent variables for propensity score calculation. The matching was performed in a 1:1 ratio, with the nearest‐neighbor method without replacement. Finally, a total of 670 patient pairs were matched after calculation.

After propensity score matching, the preexisting statistical differences between the groups were well‐balanced, except for the presence of more frequent spinal lesions in the standard‐dose RT group (*P* = 0.031). The median OS for the standard‐dose RT and dose‐escalated RT groups were 6.27 and 8.48 months (*P* < 0.001), respectively (Table 1). Significantly improved 1‐ and 2‐year OS rates were achieved in the dose‐escalated RT group, as compared to the standard‐dose RT group (40.7% and 34.2% vs. 23.7% and 19.1%; *P* < 0.001; Table 1). In the multivariate analysis after propensity score matching, the RT dose retained its independent association with OS (HR, 0.887, 95% CI, 0.737–0.951; *P* = 0.011; Table 2).

### Subgroup analyses

**Figure 2:**
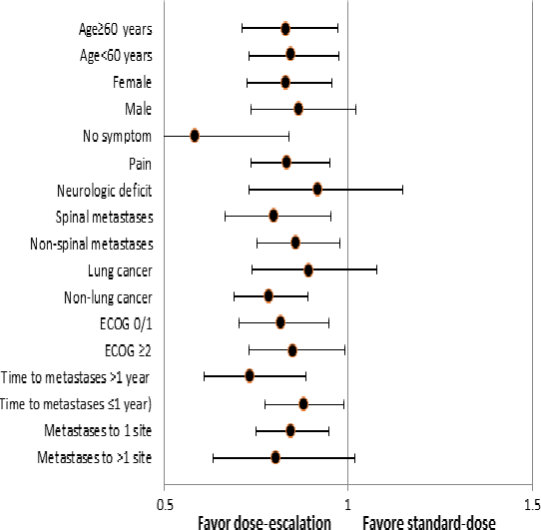
The forest plot: impact of the radiotherapy (RT) dose on overall survival by subgroups.

We then investigated whether dose‐escalated RT could be more effective in specific patient subgroups. We found that dose‐escalated RT exerted more favorable effects on OS in the following three subgroups: patients with non‐lung cancer, patients without multiple metastases, and patients without symptoms (Table 3; Figure 2).

**Table 3:** Multivariate Cox regression analysis of overall survival in subgroups

	Deaths/patients	Hazard ratio (95% CI)	*P*		Deaths/patients	Hazard ratio (95% CI)	*P*
Age (years) <60				Lung cancer			
Standard‐dose RT	407/447	1		Standard‐dose RT	428/443	1	
Dose‐escalated RT	305/340	0.832 (0.712–0.972)	0.021	Dose‐escalated RT	158/164	0.894 (0.740–1.079)	0.241
Age (years) ≥60				Non‐lung cancer			
Standard‐dose RT	654/678	1		Standard‐dose RT	633/682	1	
Dose‐escalated RT	302/330	0.844 (0.731–0.976)	0.022	Dose‐escalated RT	449/506	0.785 (0.691–0.891)	<0.00
Female				ECOG 0–1			
Standard‐dose RT	624/650	1		Standard‐dose RT	473/515	1	
Dose‐escalated RT	341/370	0.834 (0.726–0.958)	0.012	Dose‐escalated RT	322/354	0.817 (0.705–0.948)	0.008
Male				ECOG 2–4			
Standard‐dose RT	437/475	1		Standard‐dose RT	588/610	1	
Dose‐escalated RT	266/300	0.867 (0.737–0.989)	0.037	Dose‐escalated RT	285/316	0.851 (0.731–0.991)	0.038
Absence of symptoms				Time to metastases >1 year			
Standard‐dose RT	70/72	1		Standard‐dose RT	265/283	1	
Dose‐escalated RT	51/61	0.581 (0.402–0.840)	0.004	Dose‐escalated RT	220/252	0.734(0.610–0.885)	<0.00
Pain				Time to metastases ≤1 year)			
Standard‐dose RT	692/735	1		Standard‐dose RT	796/842	1	
Dose‐escalated RT	423/469	0.836 (0.736–0.950)	0.006	Dose‐escalated RT	387/418	0.880 (0.775–0.990)	0.049
Neurological deficits				Metastases to 1 site			
Standard‐dose RT	299/318	1		Standard‐dose RT	220/270	1	
Dose‐escalated RT	133/141	0.918 (0.733–1.15)	0.457	Dose‐escalated RT	120/168	0.844 (0.750–0.949)	0.005
Spinal metastases				Metastases to >1 site			
Standard‐dose RT	813/863	1		Standard‐dose RT	841/855	1	
Dose‐escalated RT	340/368	0.800 (0.668–0.955)	0.015	Dose‐escalated RT	487/502	0.805 (0.635–1.020)	0.073
Non‐spinal metastases							
Standard‐dose RT	248/262	1					
Dose‐escalated RT	267/302	0.859 (0.754–0.978)	0.022				

RT, radiotherapy; HR, hazard ratio; CI, confidence interval; ECOG, Eastern Cooperative Oncology Group.

**Figure 3:**
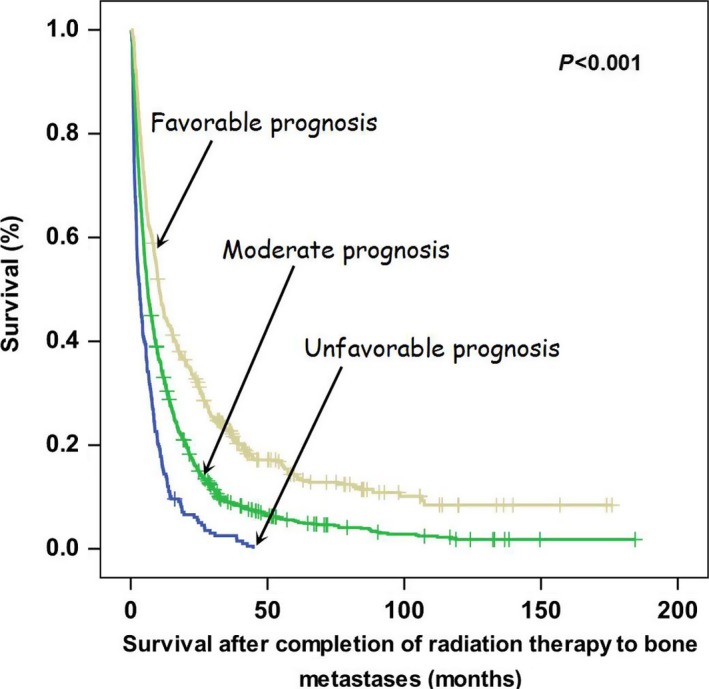
Kaplan–Meier plots of overall survival in patients with favorable prognosis, moderate prognosis, and unfavorable prognosis.

**Figure 4:**
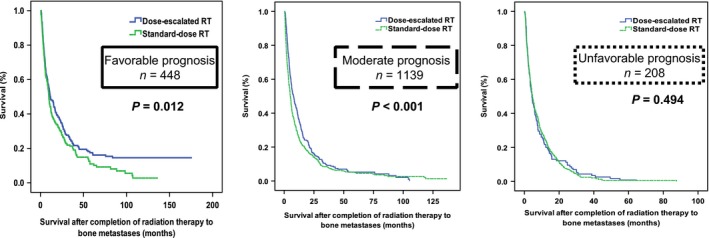
Kaplan–Meier plots of overall survival according to the radiotherapy (RT) dose in the three subgroups of patients with favorable prognosis, moderate prognosis, and unfavorable prognosis.

These characteristics were also independently good prognostic factors for OS. After combining with performance status ECOG 0–1, the four characteristics were then used to categorize the entire study cohort into subgroups, as follows: patients expected to have a favorable prognosis (with 3–4 favorable prognostic factors), patients expected to have a moderate prognosis (with 1 or 2 favorable prognostic factors), and patients expected to have an unfavorable prognosis (without any favorable prognostic factor, Figure 3; Figure S1). Dose‐escalated RT showed better OS rates in patients with favorable (*P* = 0.012) or moderate prognosis (*P* < 0.001) as compared to those treated with standard‐dose RT. In contrast, patients with unfavorable prognosis did not show a better OS when treated with dose‐escalated RT (*P* = 0.880; Figure 4). The results of multivariate analysis indicated that the radiation dose was independently associated with OS in patients with favorable prognosis (HR, 0.784; 95% CI, 0.635–0.968; *P* = 0.024) and in those with moderate prognosis (HR, 0.808; 95% CI, 0.708–0.922 *P* = 0.002). However, no such association was evident in patients with unfavorable prognosis (Table 4).

**Table 4:** Multivariate Cox regression analysis of overall survival in the subgroups of patients have different prognosis

	Number of patients	Standard‐dose RT (*n* = 1125; 63%)	Dose‐escalated RT (*n* = 670; 37%)	HR (95% CI)	*P*
Expected favorable prognosis	448	202/223	183/225	0.784 (0.635–0.968)	0.024
Expected moderate prognosis	1139	711/753	365/386	0.808 (0.708–0.922)	0.002
Expected unfavorable prognosis	208	148/149	59/59	1.048 (0.772–1.424)	0.763

RT, radiotherapy; HR, hazard ratio; CI, confidence interval.

The dose‐escalated RT group demonstrated a significantly better OS than the standard‐dose RT group in both the spiral and nonspiral BM subgroup analyses (*P* = 0.015, *P* = 0.022; Table 3).

## Discussion

The results of our retrospective study indicate that dose‐escalated RT is associated with a better OS than standard‐dose RT in patients with BM from solid tumors. Although the two study groups exhibited significant baseline differences in terms of the time to BM, initial symptoms, performance status, and primary tumor location, the radiation dose retained its independent significance as a predictor of OS in the multivariate analysis. After propensity score matching, the baseline characteristics were appropriately balanced. Nevertheless, the radiation dose was still a predictor of OS. These findings are consistent with those shown by Rade et al. 14, who showed that dose‐escalated RT (EQD_2Gy_ = 39–40 Gy) significantly improved the OS, as compared to standard‐dose RT (EQD_2Gy_ = 32.5 Gy; 30 Gy administered in 10 fractions) in 382 patients with spinal cord compression caused by metastases.

The 2‐year local control rate was significantly better in dose‐escalated RT than in standard‐dose RT group, 95.2% and 90.3%, respectively. Our result is consistent with that of a previous study by Yamada et al. 9, who found that higher RT doses delivered to the metastatic spinal lesions resulted in better local control (24 Gy vs. <24 Gy, *P* = 0.03; ≥23 Gy vs. <23 Gy, *P* = 0.04). Additionally, Laufer et al. 8 reported a superior local control in patients who received a high‐dose RT schedule (27 Gy in 3 fractions), compared with those who received a low‐dose schedule (30 Gy in 5 or 6 fractions), with 1‐year local relapse rates of 4% and 22%, respectively. Another study demonstrated that remineralization and recalcification were significantly better with the schedule involving 30 Gy administered in 10 fractions than in the schedule involving 8 Gy administered in a single fraction 15.

In the subgroup analysis, we identified three independent favorable prognostic factors (non‐lung cancer, absence of multiple metastases, and absence of symptoms) that predicted not only the OS, but also the response to dose‐escalated RT. We assumed that the benefits of dose‐escalated RT on OS are markedly greater when the life expectancy is higher. The patient's performance status was added to further stratification because the role of the performance status as a prognostic factor for cancer patients is supported by a previous study 16. As predicted, patients who were expected to have a favorable prognosis (median OS, 11.79 months) were significantly influenced by the administration of dose‐escalated RT (HR, 0.784; *P* = 0.024). However, patients who were expected to have an unfavorable prognosis (median OS, 4.03 months) did not significantly benefit from dose‐escalated RT (*P* = 0.762). These findings are consistent with those of a previous report, which showed that the escalation of the radiation dose to an EQD_2Gy_ of 39–40 Gy did not improve local control or OS, as compared to an EQD_2Gy_ of 32.5 Gy (30 Gy administered in 10 fractions) in patients with spinal cord compression due to BM 17. In contrast, dose‐escalated RT yielded better local control and OS in patients with metastatic spinal cord compression who showed a relatively favorable survival 14, 18.

The present study had certain limitations. First, the baseline characteristics of patients in the dose‐escalated and standard‐dose groups were not suitably balanced. For example, differences were observed in terms of the time to BM, performance status, and primary tumor location. Even after using propensity score matching to balance the statistical differences between the groups, our study remains prone to selection bias, and hence, we cannot exclude the presence of residual confounding factors that were not accounted for. Consequently, our current findings should be considered as hypothesis‐generating and require independent confirmation in larger, well‐designed longitudinal studies.

## Conclusions

Dose‐escalated RT, with doses >30 Gy administered in 10 fractions, was significantly associated with better OS in patients with BM from solid malignancies. The favorable effects of dose‐escalated RT on OS were particularly evident in patients with non‐lung cancer, those without multiple metastases, those without symptoms, and those with favorable prognosis.

## Conflict of Interest

None declared.

## Supporting information


**Table S1.** Relationship between radiotherapy doses and primary cancer location.
**Figure S1.** Patient stratification into different subgroups based on the different prognosis.
