# Supplementary material for: Dose‐escalated radiation therapy is associated with better overall survival in patients with bone metastases from solid tumors: a propensity score‐matched study
Source: Cancer Med. 2017 Aug 15;6(9):2087–97. doi: 10.1002/cam4.1150 (PMC5603838; doi:10.1002/cam4.1150)
Supplement: Supplementary file 1 — Table S1. Relationship between radiotherapy doses and primary cancer location. Figure S1. Patient stratification into different subgroups based on the different prognosis. [file CAM4-6-2087-s001.doc]

|  | Standard-dose RT  n=1125 (63%) | Dose-escalated RT  n=670 (37%) | Entire cohort  n=1795 (100%) | P |
| --- | --- | --- | --- | --- |
| Primary cancer |  |  |  | <.000^a^ |
| Lung, n (%) | 443 (39.4%) | 164 (24.5%) | 607 (33.8%) |  |
| Urinary tract, n (%) | 193 (17.2%) | 103 (15.4%) | 296 (16.5%) |  |
| Breast, n (%) | 122 (10.8%) | 120 (17.9%) | 242 (13.5%) |  |
| Gastrointestinal, n (%) | 111 (9.9%) | 99 (14.8%) | 210 (11.7%) |  |
| Unknown site, n (%) | 104 (9.2%) | 30 (4.5%) | 134 (7.5%) |  |
| Colorectal, n (%) | 60 (5.3%) | 43 (6.4%) | 103 (5.7%) |  |
| Gynecologic, n (%) | 20 (1.8%) | 33 (4.9%) | 53 (3.0%) |  |
| Oral cavity, n (%) | 23 (2.0%) | 29 (4.3%) | 52 (2.9%) |  |
| Nasopharyngeal, n (%) | 25 (2.2%) | 23 (3.4%) | 48 (2.7%) |  |
| Sarcoma, n (%) | 10 (0.9%) | 9 (1.3%) | 19 (1.1%) |  |
| Pharyngolaryngeal, n (%) | 8 (0.7%) | 10 (1.5%) | 18 (1.0%) |  |
| Skin, n (%) | 6 (0.5%) | 7 (1.0%) | 13(0.7%) |  |
| ^a^Two-tailed χ^2^ test. Abbreviation: RT, radiotherapy. | | | | |

**Supplementary Table 1. Relationship between radiotherapy doses and primary cancer location**

**
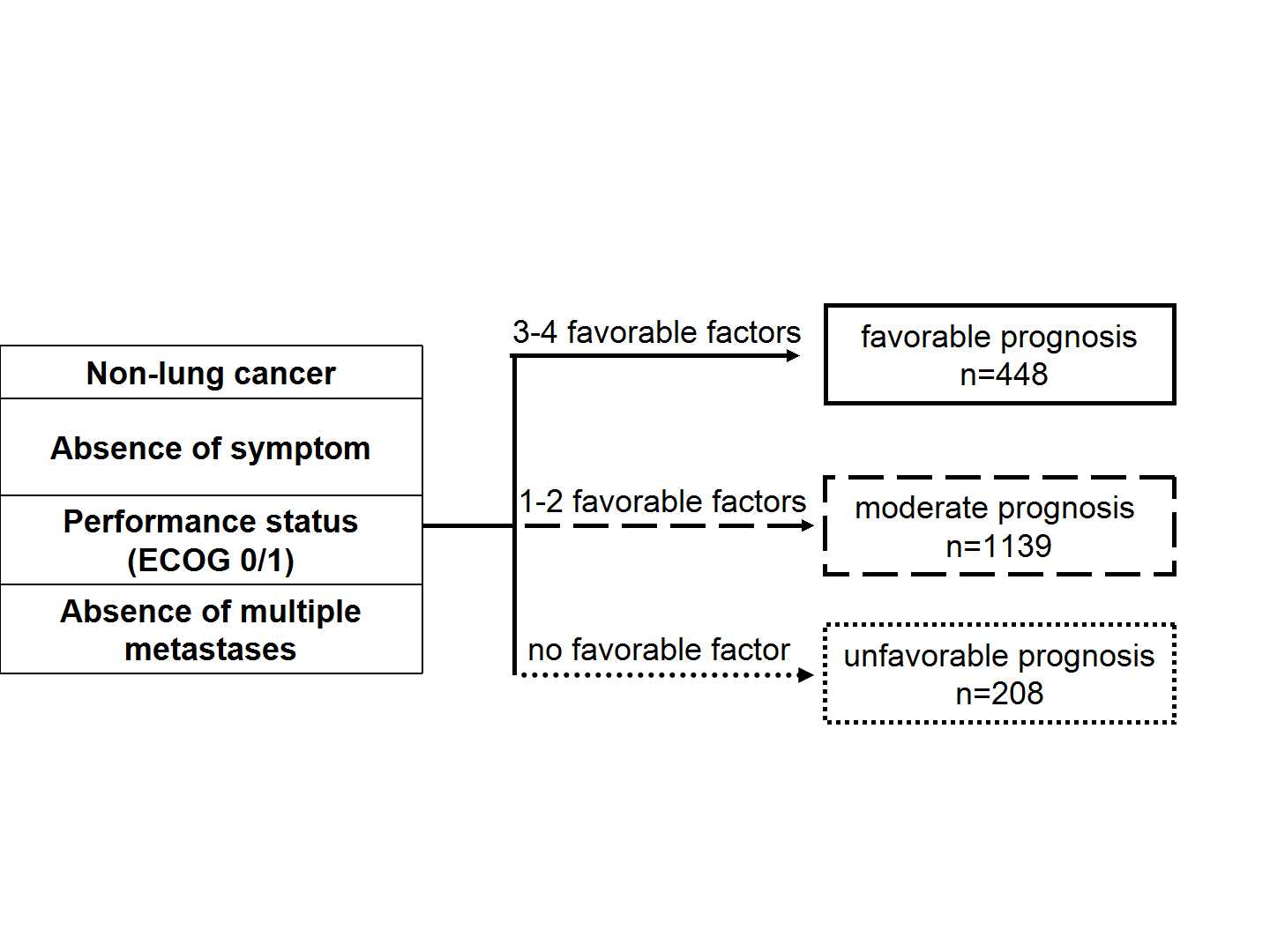
**

**Supplementary figure 1. Patient stratification into different subgroups based on the different prognosis**

*Footnote: Patients with favorable prognosis had 3 or 4 favorable factors; patients with moderate prognosis had 1 or 2 favorable factors; and patients with unfavorable prognosis had no favorable factor.*
